# Interest of pregnant women in the use of SMS (short message service) text messages for the improvement of perinatal and postnatal care

**DOI:** 10.1186/1742-4755-9-9

**Published:** 2012-08-06

**Authors:** Gabriela Cormick, Natalie A Kim, Ashlei Rodgers, Luz Gibbons, Pierre M Buekens, José M Belizán, Fernando Althabe

**Affiliations:** 1 Department of Mother & Child Health Research, Institute for Clinical Effectiveness and Health Policy (IECS), Dr. Emilio Ravignani 2024, Buenos Aires, C1414CPV, Argentina; 2 School of Public Health and Tropical Medicine, Tulane University, 1440 Canal St., Ste. 2430, New Orleans, LA, 70112-2705, USA

**Keywords:** mHealth, Mobile health, SMS text messages, Perinatal, Prenatal, Healthcare, Argentina

## Abstract

**Background:**

Mobile health (mHealth) is emerging as a useful tool to improve healthcare access especially in the developing world, where limited access to health services is linked to poor antenatal care, and maternal and perinatal mortality.

The objective of this study is to 1) understand pregnant women’s access and usage of cell phones and 2) survey the health information needs and interests in a population attending public hospitals and health centers of two cities in Argentina. This information is not available and it is the basis to develop a strategy for improving maternal care via cell phones.

**Methods:**

Questionnaires were verbally administered to pregnant women who were attending an antenatal care visit in community health centers and public hospitals in Rosario, Santa Fe and Mercedes, Corrientes. Participants were 18 years of age or older and had previously given birth. The data obtained was qualitative and analyzed using SPSS version 18.

**Results:**

A total of 147 pregnant women meeting inclusion criteria (Rosario: 63; Mercedes: 84) were approached and verbally consented to participate. The average age was 29.5 years, most lived in urban areas (89%) with a mean travel time of 43.4 minutes required to get to the health center and 57.3 minutes to get the hospital.

Ninety-six percent of women (n = 140) responded that they would like to receive text messages and cell phone calls with information regarding prenatal care, although the topics and period of time to receive information varied greatly.

**Conclusions:**

Considering the vast majority of the interviewed women had access to and were interested in receiving text messages and calls with educational information regarding pregnancy and infant health, pregnant women in Argentina could benefit from such an mHealth program. The low access to Internet suggests it is not an option for this population; however, this cannot be assumed as representative of the country’s situation.

To retain active participation, other forms of health communication, such as a 2-way text message systems or toll-free numbers, could be considered in the future. Cost of use and implementing these options should be studied.

## Background

Mobile health (mHealth) is emerging as a useful tool to improve access to health especially in areas with a limited health care workforce, limited financial resources, and a high burden of disease such as the developing world [1]. Short message service (SMS) has the potential to affect behavioral change due to its efficiency, low cost, and capability of disseminating health information to hard-to-reach populations [2].

Economically disadvantaged populations commonly use health services less. A review on access to health in low- and middle- income countries shows a lack of proper evidence on ways to reduce limiting barriers; especially those on the demand end related to the lack of education and information, the cost of access, and cultural issues [3].

Limited access to health services is linked to low antenatal care and maternal and perinatal mortality [4]. mHealth could be a very useful strategy for low- and medium- income countries to improve antenatal care. as it is suggested by programs implemented in Zanzibar, Tanzania (Wired Mothers) [5], the United States (Text4Baby) [6], and Serbia (Beba Dolazi) [7] in which gestational period specific text messages are sent to subscribed women to provide educational material, however the impact of this programs is still in evaluation [8-10]. We proposed this study as a crucial initial step to design and test a mHealth strategy to address this problem in Argentina.

The objective of this study was to understand pregnant women’s level of access to cell phones, behaviors of cell phone usage, and information needs and interests in a population attending public hospitals and health centers of two cities of Argentina. This information is not available in the literature and is the basis to develop a strategy using cell phones for the improvement of maternal care.

## Methods

Questionnaires were administered to assess knowledge, attitude and behaviors surrounding mobile phone and text message use among pregnant women in community health centers and public hospitals in Rosario, Santa Fe (Maternidad Martin and Roque Saenz Peña Hospital) and Mercedes, Corrientes (Las Mercedes Hospital, Axel Verón Health Center, San Francisco Health Center, and San Martin Health Center). Rosario is a city of around 1.5 million inhabitants while Mercedes is a city of around 40,000 inhabitants surrounded by a wide rural area. The two sites in Rosario were selected based on that they are the biggest hospitals in the city assisting around 60% of its public deliveries. In Corrientes, the selection was made based on a representation of the various sites providing antenatal care, a unique hospital and 3 peripheral health centres.

The public health sector in Argentina provides free health care to all population; however it is mainly used by the population whose health care is not provided by labor union insurance funds or the private sector [11]. This group represented 38% of the population during the 2010 census and is identified as the less socioeconomically advantaged. This group represents more than 48% of the population in province of Corrientes and around 32% in the province of Santa Fe [12,13]. On average, 14% of this population live in rural areas, with some northern provinces averaging more than 25%, and presumably face more difficulties in accessing health services [14].

The inclusion criteria of the study required the participants to be pregnant women, at least 18 years, who had previously given birth to a live fetus. We decided to include only women who had previously given birth to a live fetus as some questions relate to late pregnancy or postpartum experiences, which women in their first pregnancy would not have had. Pregnant women included in this study were able to recall their previous experience to answer the questions of this study.

Sampling was performed by convenience. Interviewers recruited all eligible women in each site until completing the sample number. As Mercedes had more centers all women included the last day exceeded the final sample number. All eligible participants were verbally asked for informed consent in Spanish, which was approved by the Institutional Review Board of Tulane University and the Ethics Committee of the Center of Medical Education and Clinical Investigations in Buenos Aires, Argentina.

During prenatal clinic hours, research personnel approached patients in waiting rooms prior to patients’ appointments. Subjects were taken to a quiet area in the waiting room and asked to verbally answer the questionnaires, which took an average of 20 minutes total. This interview included demographic characteristics, use of technology and willingness to receive information via mobile phone. We also asked about travel time to the health centre and to the hospital. Interviewers received training on interviewing techniques and information regarding mHealth. The completed questionnaires were stored in binders in the nurse’s station until collected by head research staff.

Data was entered into Excel spreadsheets and means and standard deviations were analyzed using SPSS version 18. Sample size of 60 subjects in each location was estimated assuming 80% of cell phones availability with a +/- 10% error in the estimation and a confidence level of 95%.

## Results

A total of 147 pregnant women meeting the inclusion criteria, 63 in Rosario and 84 in Mercedes, were approached and verbally consented in hospital and health center waiting rooms. None of the subjects declined to participate, although one subject had to leave before the completion of all three questionnaires.

Table 1 provides the demographic characteristics of women interviewed. The average age was 29.5 years, most lived in urban areas (89%) with a mean travel time of 43.4 minutes required to get to the health center and 57.3 minutes to get to the hospital, although this varied greatly among the sites. Twenty-five of them (17.1%) reported not having completed primary school.

**Table 1:** Demographic Information

**Characteristics**		**Mercedes (n = 83)**	**Rosario (n = 63)**	**All (n = 146)**
		**n (%)**	**n (%)**	**n (%)**
Age (years) (the study included only women over 18)	Less than 20	2 (2.4)	2 (3.2)	4 (2.7)
	20 to 34	55 (66.3)	56 (88.9)	111 (76.0)
	35 and more	26 (31.3)	5 (7.9)	31 (21.2)
Country of birth	Argentina	79.0 (95.2)	60.0 (95.2)	139.0 (95.2)
	Other	4.0 (4.8)	3.0 (4.8)	7.0 (3.5)
Area of residence	Urban	71.0 (85.5)	59.0 (93.7)	130.0 (89.0)
	Rural	8.0 (9.6)	2.0 (3.2)	10.0 (6.8)
	No response	4.0 (4.8)	2.0 (3.2)	6.0 (4.1)
Travel time required to get to the health center (in minutes)	Less than 30	45 (54.9)	51 (81.0)	96(66.2)
	30 to 59	15 (18.3)	7 (11.1)	22 (15.2)
	60 to 119	8 (9.8)	4 (6.3)	12 (8.3)
	120 or more	14 (17.1)	1 (1.6)	15 (10.3)
Travel time required to get to the hospital (in minutes)	Less than 30	14 (16.8)	25 (39.7)	39 (26.7)
	30 to 59	28 (34.1)	33 (52.4)	61 (41.8)
	60 to 119	26 (31.7)	4 (6.3)	30 (20.5)
	120 or more	14 (17.4)	1 (1.6)	15 (10.3)
	Missing	1 (1.2)	0 (0.0)	1 (0.7)
Number of people in the household	Less than 5	31(37.3)	34 (54.0)	65 (44.5)
	5 or more	52 (62.7)	29 (46.0)	81 (55.5)
Number of previous births	1-2	43 (51.8)	46 (73.0)	89 (61.0)
	3-4	17 (20.5)	14 (22.2)	31 (21.2)
	5 or more	23 (27.7)	3 (4.8)	26 (17.8)
Number of children	1-2	44 (53.0)	46 (73.0)	90 (61.6)
	3 to 4	16 (19.3)	14 (22.2)	30 (20.5)
	5 or more	23 (27.7)	3 (4.8)	26 (17.8)
Women education level	Less than primary school	5 (6.0)	0.0 (0.0)	5.0 (3.4)
	Primary education, incomplete	15 (18.1)	5 (7.9)	20 (13.7)
	Primary education, complete	22 (26.5)	16 (25.4)	38 (26.0)
	Secondary education, incomplete	25 (30.1)	19 (30.2)	44 (30.1)
	Secondary education, complete	13 (15.7)	18 (28.6)	31 (21.2)
	Higher education, incomplete	1 (1.2)	3 (4.8)	4 (2.7)
	Higher education, complete	2 (2.4)	2 (3.2)	4 (2.7)

Of 146 subjects, 136 women (93.2%) were using cell phones at the time of the interview; fifty-six of whom (38.4%) have had the same phone number for more than one year (Table 2). The majority (74.7%) was using a prepaid cell phone method versus a contracted plan. The average age of first cell phone ownership was 22.2 years with a standard deviation of 5.7 years. Very few subjects (n = 6; 4.1%) had and used the internet on their cell phones while the majority (61%) did not use internet regularly neither on cell phones or on computers.

**Table 2:** Use of mobile phones and internet

		**Mercedes (n = 83)**	**Rosario (n = 63)**	**All (n = 146)**
		**n (%)**	**n (%)**	**n (%)**
Currently have a cell phone	Yes	79 (95.2)	57 (90.5)	136 (93.2)
	No	4 (4.8)	6 (9.5)	10 (6.8)
Length of time having cell phone	<6 months	9 (10.8)	16 (25.4)	25 (17.1)
	6 months to less than 1 year	9 (10.8)	4 (6.3)	13 (8.9)
	1 year to less than 2 years	10 (12)	15 (23.8)	25 (17.1)
	2 years or more	49 (59)	26 (41.3)	75 (51.4)
	Don’t know/No response	6 (7.2)	2 (3.2)	8 (5.4)
Cell phone plan	Contract	10 (12)	23 (36.5)	33 (22.6)
	Prepaid	69 (83.1)	40 (63.5)	109 (74.7)
	Don’t know	4 (4.8)	0 (0)	4 (2.7)
Age at first cell phone	Less than15	0 (0.0)	1 (1.6)	1 (0.7)
	15 to 1920	23 (27.7)	29 (46.0)	52 (35.6)
	20 to <35	52 (62.7)	30 (47.6)	82 (56.2)
	35 or more	3 (3.6)	2 (3.2)	5 (3.4)
	Missing	5 (6.0)	1 (1.6)	6 (4.1)
Changed cell number in the last 12 months	Never	22 (26.5)	34 (54)	56 (38.4)
	1 time	26 (31.3)	13 (20.6)	39 (26.7)
	2 times	22 (26.5)	8 (12.7)	30 (20.5)
	3 times	3 (3.6)	4 (6.3)	7 (4.8)
	4 or more times	5 (6)	4 (6.3)	9 (6.2)
Use of internet on the cell phone	Yes	3 (3.6)	3 (4.8)	6 (4.1)
	No access	54 (65.1)	31 (49.2)	85 (58.2)
	No response	26 (31.3)	29 (46.0)	55 (37.7)
Location of the computer for internet use	Home	17 (20.5)	20 (31.7)	37 (25.3)
	Call centre	5 (6.0)	2 (3.2)	7 (4.8)
	Work/school	3 (3.6)	3 (4.8)	6 (4.1)
	Friend/Family	1 (1.2)	2 (3.2)	3 (2.1)
	No regular use of internet	53 (63.9)	36 (57.1)	89 (61.0)
	No response	4 (4.8)	0 (0)	4 (2.7)

One hundred and forty (96%) responded that they would like to receive text messages with information regarding prenatal care (Table 3). While women consistently showed interest in receiving the informational text messages, the time at which they would want to begin receiving text messages varied greatly (before pregnancy: 11%; month 1: 33%; month 3: 23%; month 6: 14%; last month of pregnancy: 14%). One hundred and thirty three women (91%) responded with interest in receiving informational text messages postpartum; again, for varying amounts of time (up to 3 months: 25%; up to 6 months 38%; up to 12 months: 17%; more than 12 months: 7%). More than half of them preferred to receive text messages with a frequency of once a week. An equally large percentage of women were interested in receiving phone calls with similar prenatal (87%) and postnatal (87%) educational information.

**Table 3:** Willingness to receive health information via mobile phone or internet

**Text messages**		**Mercedes (n = 83)**	**Rosario (n = 63)**	**All (n = 146)**
		**n (%)**	**n (%)**	**n (%)**
Willingness to receive SMS during pregnancy	Yes	78 (94.0)	62 (98.4)	140 (95.9)
	No	2 (2.4)	1 (1.6)	3 (2.1)
	Don’t know	3 (3.6)	0 (0.0)	3 (2.1)
Time to begin receiving SMS	Before pregnancy	9 (10.8)	7 (11.1)	16 (11)
	From month 1	39 (47)	9 (14.3)	48 (32.9)
	From month 3	16 (19.3)	17 (27)	33 (22.6)
	From month 6	6 (7.2)	14 (22.2)	20 (13.7)
	From month 9 (before birth)	6 (7.2)	15 (23.8)	21 (14.4)
	Don’t know/No response	7 (8.4)	1 (1.6)	8 (5.4)
Willingness to receive SMS, post-partum	Yes	75 (90.4)	58 (92.1)	133 (91.1)
Period to receive SMS, post-partum	0 to 2 months	13 (15.7)	23 (36.5)	36 (24.7)
	3 to 5 months	40 (48.2)	16 (25.4)	56 (38.4)
	6 to 11 months	12 (14.5)	13 (20.6)	25 (17.1)
	12 months or more	4 (4.8)	6 (9.5)	10 (6.8)
	Don’t know/No response	14 (16.8)	5 (7.9)	19 (13)
Preferred time of the day for receiving SMS	Morning (8 am - before12pm)	31 (37.3)	21 (33.3)	52 (35.6)
	Afternoon (12 pm - before 4 pm)	11 (13.3)	7 (11.1)	18 (12.3)
	Evening (4 pm - before 8 pm)	7 (8.4)	10 (15.9)	17 (11.6)
	Any time	30 (36.1)	23 (36.5)	53 (36.3)
	Don’t know/No response	4 (4.8)	2 (3.2)	6 (4.1)
Preferred frequency to receive SMS	1 per week	40 (48.2)	37 (58.7)	77 (52.7)
	3 per week	31 (37.3)	13 (20.6)	44 (30.1)
	5 per week	2 (2.4)	1 (1.6)	3 (2.1)
	7 per week	6 (7.2)	9 (14.3)	15 (10.3)
	Don’t know/No response	4 (4.8)	3 (4.8)	7 (4.8)
Preferred pregnancy information	Activities/things to avoid	76 (91.6)	59 (93.7)	135 (92.5)
	When to call a doctor	77 (92.8)	56 (88.9)	133 (91.1)
	Diet	70 (84.3)	62 (98.4)	132 (90.4)
	Appointment reminders	79 (95.2)	40 (63.5)	119 (81.5)
	Family planning	73 (88)	45 (71.4)	118 (80.8)
	What to expect at various stages of pregnancy	70 (84.3)	46 (73)	116 (79.5)
	Mental health	73 (88)	41 (65.1)	114 (78.1)
	Physical activity	61 (73.5)	45 (71.4)	106 (72.6)
	Pregnancy & delivery courses	69 (83.1)	24 (38.1)	93 (63.7)
Preferred infant information	Baby skin care	77 (92.8)	62 (98.4)	139 (95.2)
	Diet	74 (89.2)	59 (93.7)	133 (91.1)
	Lactation	76 (91.6)	57 (90.5)	133 (91.1)
	When to call a doctor	74 (89.2)	56 (88.9)	130 (89)
	Appointment reminders	79 (95.2)	42 (66.7)	121 (82.9)
**PHONE CALLS**				
Willingness to receive phone calls	Yes	78 (94.0)	49 (77.8)	127 (87.0)
during pregnancy	No	4 (4.8)	12 (19.0)	16 (11.0)
	Don’t know	1 (1.2)	2 (3.2)	3 (2.1)
Time to begin receiving calls during pregnancy	Before pregnancy	7 (8.4)	4 (6.3)	11 (7.5)
	Month 1	41 (49.4)	5 (7.9)	46 (31.5)
	Month 3	15 (18.1)	14 (22.2)	29 (19.9)
	Month 6	6 (7.2)	10 (15.9)	16 (11)
	Month 9 (before birth)	5 (6)	14 (22.2)	19 (13)
	Don’t know/No response	6 (7.2)	0 (0)	24 (16.4)
Willingness to receive phone calls post-partum	Yes	78 (94)	49 (77.8)	127 (87)
	No	4 (4.8)	12 (19)	16 (11)
	Don’t know	1 (1.2)	2 (3.2)	3 (2.1)

Overall, the women were interested in finding out all information regarding pregnancy and newborn health, especially including prenatal (90%) and infant (91%) dietary information, activities/things to avoid during pregnancy (92%), when to call a doctor during pregnancy (91%), lactation counseling (91%), and infant skin care (95%). The topics with lowest interest included physical activity during pregnancy (73%) and pregnancy/delivery course information (64%).

Even though we cannot statistically compare the results obtained from both cities, the sample from Mercedes included more women who were older than 35 years (31.3% compared to 7.9% in Rosario) and that took more than 120 minutes to reach the health centre or the hospital (17% compared to 1.6% in Rosario).

A similar number of women from both cities reported having a cell phone, however a larger number of women in Mercedes reported having a cell phone for more than 2 years (59% compared to 41.3) and fewer reported not having changed the number in the last year (26.5% compared to 54%).

The preferred information to be received by SMS was similar in both groups, although more women in Mercedes were interested in receiving appointment reminders (95.2% compared to 63.5%) and mental health advice (88% compared to 65.1%) than in Rosario. More women from Mercedes than in Rosario agreed to receive phone calls during pregnancy and post- partum (94.0% compared to 77.8%).

## Discussion

The vast majority of the interviewed women had access to and was open to receive SMS text messages and cell phone calls with educational information regarding pregnancy and infant health. According to our findings, women would be willing to be enrolled in a mHealth one-way text-messaging program at their antenatal visit and receive information via text message regarding everything from prenatal/infant diet, to lactation information, to infant skin care. The most preferred approach was text messages sent out one or three times a week. Ideally, women should be able to choose when and how frequently they would receive text messages. Since a majority of women own cell phones, and report to be interested in receiving educational information via SMS text message, pregnant women in Argentina could benefit from a mHealth program. Owing to the low access to Internet via cell phone and computer, it did not appear to be a good option for communicating with this population.

The results come from two distinct regions of Argentina: Rosario, one of the largest cities of the country, and Mercedes, a small city located in one of the poorest provinces of Argentina. However, we cannot assume that this sample could be representative of the entire population as it was a convenient sample of women interviewed at the health centre or hospital therefore those attending antenatal care more frequently had a greater chance of being included.

A potential drawback to implementing a text-messaging program is that it requires the recipient to have an adequate level of literacy, marginalizing groups who could potentially benefit from the intervention. In our study population this could affect around 17% of women having no or incomplete primary schooling.

The limitation of sending only 160 characters in a text message poses a challenge for healthcare providers to send detailed messages regarding the health and care of an individual. [15] User guidelines need to be established for mHealth programs to help manage privacy and security issues especially considering mobile phones are often shared among family and community members [16].

To retain active participation, other forms of health communication could be considered in the future such as a 2-way text message systems or toll-free numbers allowing women to express their questions or concerns. Cost of use and implementation of these options should be studied. The increased use of technology can help reduce health care costs by improving efficiencies in the health care system and promoting prevention through behavior change communication.

Although the use of the Internet might have a similar potential in developed countries, this is still not applicable globally [17]. Information in this study performed in two cities from a middle income country shows that the vast majority of women did not have regular access to the internet.

## Conclusion

This study shows that cell phones would be an acceptable approach to provide pregnancy and postpartum support to women of low socioeconomical level in a middle income country, since the vast majority of women interviewed had access to a cell phone and referred it as a desired and accepted mean of communication.

## Abbreviations

mHealth, Mobile health; SMS, Short message service.

## Competing interests

The authors declare that they have no competing interests.

## Authors’ contributions

GC participated in study design, data collection/analysis, and manuscript drafting. NK and AR participated in study design, IRB submission, data collection/analysis, and manuscript drafting. LG participated in statistical analysis. PB participated in study design and manuscript drafting. JB participated in study design, study coordination, data interpretation, and manuscript drafting. FA participated in study design, and manuscript drafting. All authors read and approved the final manuscript.
